# Obesity and COVID-19: what makes obese host so vulnerable?

**DOI:** 10.1186/s12979-020-00212-x

**Published:** 2021-01-04

**Authors:** Sameer Mohammad, Rafia Aziz, Saeed Al Mahri, Shuja Shafi Malik, Esraa Haji, Altaf Husain Khan, Tanvir Saleem Khatlani, Abderrezak Bouchama

**Affiliations:** 1 Experimental Medicine Department, King Abdullah International Medical Research Center/King Saud bin Abdulaziz University for Health Sciences-MNGHA, Riyadh, 11426 Saudi Arabia; 2 Government Medical College Baramulla, Baramulla, Kashmir India; 3 Biostatistics and Bioinformatics Department, King Abdullah International Medical Research Center/King Saud bin Abdulaziz University for Health Sciences-MNGHA, Riyadh, 11426 Saudi Arabia; 4 Department of Cellular Therapy, Stem Cells Unit, King Abdullah International Medical Research Center/King Saud bin Abdulaziz University for Health Sciences--MNGHA, Riyadh, 11426 Saudi Arabia

**Keywords:** Coronavirus, Covid-19, Obesity, Innate and adaptive immunity, Inflammation, Insulin resistance

## Abstract

The disease (COVID-19) novel coronavirus pandemic has so far infected millions resulting in the death of over a million people as of Oct 2020. More than 90% of those infected with COVID-19 show mild or no symptoms but the rest of the infected cases show severe symptoms resulting in significant mortality. Age has emerged as a major factor to predict the severity of the disease and mortality rates are significantly higher in elderly patients. Besides, patients with underlying conditions like Type 2 diabetes, cardiovascular diseases, hypertension, and cancer have an increased risk of severe disease and death due to COVID-19 infection. Obesity has emerged as a novel risk factor for hospitalization and death due to COVID-19. Several independent studies have observed that people with obesity are at a greater risk of severe disease and death due to COVID-19. Here we review the published data related to obesity and overweight to assess the possible risk and outcome in Covid-19 patients based on their body weight. Besides, we explore how the obese host provides a unique microenvironment for disease pathogenesis, resulting in increased severity of the disease and poor outcome.

## Introduction

COVID-19 disease is caused by a novel coronavirus (SARS-COV-2) that emerged in the Wuhan province of china [1, 2]. The first documented human infection was reported in Dec 2019 and since then, the disease has spread at an unprecedented speed and magnitude to become the greatest healthcare concern of the twenty-first century [3–5]. Even with the implementation of major interventions to contain the spread of the disease, COVID-19 has progressed worldwide resulting in significant morbidity and mortality [6–8]. As of Oct 5, 2020, the total number of infected patients stands at 35 million resulting in more than a million deaths. As a consequence, intense efforts are on to understand the epidemiology and pathobiology of this disease. The global fatality rate of CoVID-19 is ~ 3%, although great differences exist with some countries (France and the United Kingdom) recording a high death rate of ~ 10% and others (India, Israel, Russia) reporting less than 2% mortality rates [9–14]. Several epidemiological studies strongly suggest a link between age and severity of the illness [15–20]. More than 75% of the deaths have been reported in patients aged 65 years or above. Also, people with co-morbidities such as diabetes, cardiovascular diseases, hypertension, and cancer have significantly higher mortality rates [21–23]. Recent studies emerging from multiple countries have shown that obesity may be an independent factor to predict the risk and outcome of COVID-19 patients [24–37]. High body mass index (BMI) has particularly been found to be a strong indicator of disease severity in patients younger than 60 years of age [38–42].

Here we summarize the available epidemiological data with a particular focus on obesity and its impact on disease severity. We also discuss possible mechanism(s) that make obese host susceptible to severe disease as a result of SARS-CoV-2 infection.

## Impact of obesity on disease severity of COVID-19 patients

There have been several reports indicating obesity to be a strong factor for becoming seriously ill with COVID-19 [43–45]. A retrospective study from Lille, France analyzed the relationship between body mass index BMI and requirement for invasive mechanical ventilation (IMV) in 124 consecutive patients admitted in intensive care for SARS-COV-2. Out of 124 patients, 84 (75.8%) were obese (BMI > 30 kg/m^2^), indicating a high incidence of obesity among patients admitted to intensive care for SARS-COV-2 [46]. When compared to ICU admissions the previous year for the severe acute pulmonary condition in the same institution, the distribution of BMI categories was strikingly different in patients admitted with COVID-19. Patients admitted with non-SAR-COV-2 conditions showed a lower prevalence of obesity (25.8%) compared to patients with SAR-COV-2. The prevalence of obesity observed in the non-SARS-COV-2 patients was similar to that observed in the general population from Nord and Pas de Calais. In contrast, sex distribution and age were not significantly different from participants in non-SARS-CoV-2 controls vs SARS-COV-2 subjects. Interestingly, obesity was also a standout factor for the requirement of Intermittent Mandatory Ventilation (IMV). Of 124 patients, 85 (68.6%) needed IMV and their BMI was higher than those who didn’t need IMV. Close to 90% of the patients with a BMI of > 35 required IMV. In a study from three hospitals in Wenzhou, China, Zheng et al. demonstrated that obesity was a major risk factor for the severity of COVID-19 in a group of patient’s metabolic associated fatty liver disease (MAFLD) [24]. The authors analyzed data from Covid-19 patients with confirmed MAFLD and showed that out of Sixty-six patients, Forty-five were overweight/obese (BMI > 25 kg/m^2^). Out of these 17 (37.8%) showed severe disease. Compared to only 2 (9.5%) non-obese patients that have severe disease. The authors concluded that obesity is a major risk factor for disease severity in COVID-19 patients having MAFLD. A recent review addressed the role of MAFLD in the outcome of COVID-19 patients [47]. Another study from Rhode Island, USA showed a strong association between obesity and disease severity. The authors analyzed data from 103 adult consecutive patients, admitted with COVID-19 to the hospital. The authors concluded that patients with extreme obesity (BMI of > 35 kg/m^2^) are at high risk of severe COVID-19. Besides, Obesity (BMI > 30 kg/m^2^) was strongly and independently associated with the use of invasive mechanical ventilation in patients with COVID-19 [48]. Similar results were shown by a study conducted by New York University health center on a large cohort of COVID 19 patients (*N* = 3615) [38]. The authors performed a prospective analysis of BMI stratified by age in COVID-19 positive symptomatic patients who showed up at the hospital. The authors showed that younger patients (Age < 60 years) with a BMI > 30 kg/m^2^ were more than twice likely to be admitted to hospital and develop critical illness compared to patients with a BMI < 30 kg/m^2^. The likelihood of admission to ICU increased to 3.6 times in patients with severe obesity (BMI ≥ 35 kg/m^2^) [38]. Another study from the same hospital with a larger sample size (*N* = 5279) showed similar results. The authors concluded that after age, obesity was the single most important factor for hospitalized patients with COVID-19 [49]. A report from the United Kingdom (a pre-print without peer-review) evaluated the fate of 16,749 hospitalized COVID-19 patients in the UK [50]. The authors concluded that Obesity was associated with a higher probability of mortality. A single center study from Italy on a cohort of 482 patients found obesity to be a strong, independent risk factor for severe diease and dealth due to COVID-19. While patients with a BMI ≥ 30 kg/m2 had a high risk for severe illness, a BMI ≥ 35 kg/m2 radically increased the risk of death [35]. Zhang *et. al*. reported that obesity predisposed young COVID-19 patients (14–45 Years of age) to the risk of significantly higher mortality [41]. Cai *et.al*. examined the association of Obesity with the severity of COVID-19 in a designated hospital in Shenzhen, China and concluded that obese patients has increased odds of progressing to severe disease due to COVID-19 [51]. Reports from other countries severely affected by the pandemic including Mexico [52], Germany [53] an Spain [54] have also found a significant association between BMI and the increasing severity of the disease and mortality due to COVID-19.

Table 1 shows the association of BMI with disease severity and mortality in COVID-19 patients from different studies. Together, these data strongly suggest obesity to be an important factor in disease severity and outcome of COVID-19 patients.

**Table 1:** Risk of poor clinical outcome in obese patients with COVID-19 infection

Reference	No. of Patients	BMI (Kg/m^2^)	Risk of critical diseaseHR/OR (95% CI)	Risk of DeathHR/OR (95% CI)
Rottoli et al. (2020) [35]	482	≥ 30	2.32 (1.31–4.09)	N/A
		≥ 35	N/A	12.1 (3.25–45.1)
Giacomelli et al. (2020) [55]	233	≥ 30	8.26 (1.41–48.29)	3.04 (1.42–6.49)
Klang et al. (2020) [56]	3406	≥ 40	N/A	5.1 (2.3–11.1)
Docherty et al. (2020) [50]	20,133	> 30	N/A	1.33 (1.19–1.51)
Caussy et al. (2020)	340	> 30	1·89 (1·33–2·53)	NA
Simonnet et al. (2020) [46]	124	≥ 35	7.36 (1.63–33.14)	NA
		30–35	3.45 (0.83–14.31)	
		25–30	1.69 (0.52–5.48)	
Lighter J et al. (2020) [38]	725	30–34	1.8 (1.2–2.7)	NA
		≥ 35	3.6 (2.5–5.3)	NA
Palaiodimos et al. (2020) [57]	200	≥ 35	NA	3.78 (1.45–9.83)
Cai et al. (2020) [51]	383	≥ 28	3.4 (1.40–2.86)	NA
Petrilli et al. (2020) [49]	1999	≥ 40	2.45 (1.78 to 3.36)	NA
		30–39	1.8 (1.47 to 2.2)	
		25–29.9	1.3 (1.07 to 1.57	
Hamer et al. (2020) [58]	760	> 30	2.05 (1.68, 2.49)	NA
Zhang et.al (2020) [41]	340	≥ 28	NA	1.354 (1.075–1.704)
Kalligeros et al. (2020) [48]	103	≥ 35	5.39 (1.13–25.64)	NA
Pettit et al. 2020 [59]	238	> 30	NA	1.7 (1.1–2.8)

*OR* Odds Ratio, *HR* Hazard ratio, *CI* Confidence Interval, *BMI* Body Mass Index

**Fig. 1:**
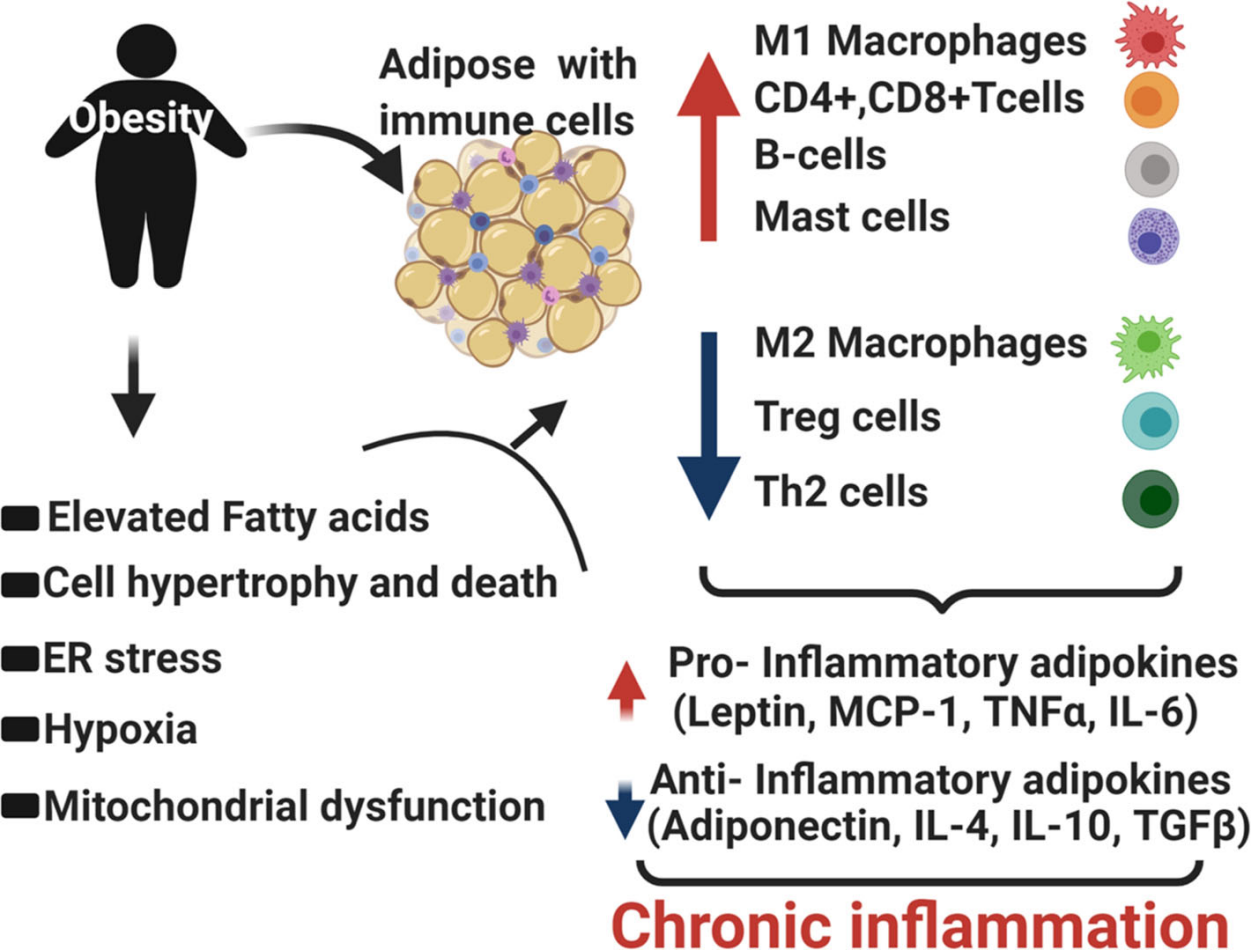
Dysregulated fatty acid metabolism, cellular hypertrophy and death, ER stress, Hypoxia and mitochondrial dysfunction, because of excess fat leads to a substantial alteration of cellular architecture of adipose tissue. This rearrangement favors a pro-inflammatory environment and perpetuates local as well as systemic inflammation

## What makes the obese host so vulnerable?

People with obesity have an increased prevalence of diseases like renal insufficiency, cardiovascular diseases, Type 2 diabetes mellitus, certain types of cancers, and a significant degree of endothelial dysfunction. These conditions are major risk factors for disease severity and mortality associated with COVID-19. This makes obesity, particularly ominous in COVID-19. However, there is enough evidence to suggest that obesity is an additional risk factor associated with worse outcomes in COVID-19 patients. Caussy et al. specifically looked at whether obesity was associated with worse outcomes in COVID-19 patients with other risk factors. The authors found that obesity remained a significant factor for poor outcome of patients having other chronic issues like Hypertension, Dyslipidemia, Type 2 diabetes, Cardiomyopathy, Chronic pulmonary diseases, and malignancy. The analysis is shown in Table 2.

**Table 2:** Odds ratios of critical COVID-19 between patients with and without obesity in Lyon University Hospital, by risk factor adjustment

	Odds ratio (95% CI) of ICU admission	*p* value*
Age, sex	2·16 (1·27–3·68)	0·0041
Hypertension, age, sex	1·93 (1·10–3·39)	0·022
Dyslipidaemia, age, sex	1·85 (1·05–3·26)	0·034
Type 2 diabetes, age, sex	1·80 (1·03–3·17)	0·040
Cardiomyopathy, age, sex	1·94 (1·11–3·40)	0·021
Chronic pulmonary disease, age, sex	2·03 (1·16–3·56)	0·013
Malignancy, age, sex	1·91 (1·09–3·34)	0·023

Table 2. Odds ratios are calculated on all patients in Lyon University Hospital with severe COVID-19. ICU = intensive care unit. COVID-19 = coronavirus disease 2019. *p-value determined using multivariable logistic regression [60]. (This table is reproduced with permission from the publisher)

Therefore, it is reasonable to assume that there are additional factors that make obese host vulnerable to severe disease and worse outcomes as a result of COVID-19 infection.

## Obesity-associated inflammation and its impact on SARS-CoV-2 infection

Until recently adipose tissue was merely considered to be an inert organ that stored energy in the form of lipids, which could be utilized in the state of fasting/starvation. However, adipose tissue is now being recognized as a key endocrine organ that secretes a plethora of factors (Adipokines, Chemokines, and Cytokines) that profoundly impact metabolism and immune system [61, 62]. Normal lean adipose is composed of a comprehensive set of immune cells that maintain a balance between pro-inflammatory and anti-inflammatory environment [63]. Excess calorie intake and/or reduced energy expenditure leads to a rapid expansion of adipose tissue to accommodate and store excess nutrients. However, obesity-induced expansion alters the function and architecture of adipose tissue and enlarged adipocytes become apoptotic and attract macrophages and other cells to form inflammatory adipose [64, 65]. Normal adipose tissue contains a population of three anti-inflammatory cell types associated with normal adipose function. T helper (Th2) cells, M-2 macrophages and regulatory T-cells (Treg) are important negative regulators of inflammation. Obesity is associated with significant alteration and abundance of immune cells in the adipose tissues with a marked decrease in Th2 cells, Treg cells, and M-2 macrophages. Instead, there is a significant increase in the abundance of pro-inflammatory cells like CD8+ T cells and M-1 macrophages [66–69]. Obese, inflamed adipose comprises of > 40% M-1 macrophages, which are the source of an array of pro-inflammatory cytokines leading to a local as well as systemic inflammation. Several other cell types like neutrophils, dendritic cells, and mast cells also contribute to inflammation by releasing several pro-inflammatory factors. The ultimate result is a state of chronic inflammation both at local as well at the systemic level [68, 70]. Inflammation is at the forefront of COVID-19 research and major complication of COVID-19 infection are directly associated with systemic inflammation [71–76]. Recent studies have indicated that disease severity and outcome of COVID-19 patients are directly associated with dysregulation of pro-inflammatory cytokines. Therefore, it is plausible to suggest that acute inflammation arising from COVID-19, may amplify existing chronic inflammation secondary to obesity and lead to more severe disease phenotype and poorer outcomes. A similar hypothesis was proposed in a recent paper by Paul MacDaragh Ryan and Noel M. Caplice [77]. The authors suggested that obese subjects have higher levels of various inflammatory signals and, are more likely to overreact to coronavirus infection. Zhang et al. analyzed 16 retrospective studies and found that inflammatory markers were positively correlated with the severity of COVID-19 [78]. Hamer *et.al*. specifically looked at the role of low inflammation in the severity of COVID-19 disease [79]. The authors found that a high rate of hospital admission in obese subjects can be partly explained by low-grade inflammation (Fig. 1).

## Cellular immune function is impaired in obesity

Several lines of evidence have strongly indicated that obesity results in significant changes in both innate and adaptive immune response and individuals with obesity are in a state of chronic and low-grade inflammation [80–82]. The overall result is a reduced immune response to infectious agents, resulting in poorer outcomes post-infection [83–86].

## Excess fat deposition disrupts lymphoid tissue architecture and integrity

Blood cells (both lymphoid and myeloid) lineages are generated from bone marrow-derived pluripotent hematopoietic stem cells. Lymphoid cells undergo further processing in the thymus to generate mature T-Lymphocytes. Mature lymphocytes reside in secondary lymphoid tissues including lymph nodes and spleen, where they take part in immune surveillance and wait for activation by pathogens. Therefore, any change in the lymphoid tissue architecture can adversely affect its functioning resulting in an alteration in the distribution of immune cell populations, impaired T cells activity, and diminished immune defense. Interestingly, Obesity and metabolic syndrome have a profound impact on the functioning of lymphoid tissue [87, 88]. Ectopic lipid deposition in tissues other than adipose is a hallmark of obesity and this is not restricted to metabolic tissues. Several studies have reported that obesity leads to increased lipid deposition in primary lymphoid organs (bone marrow and thymus). Excess lipid deposition in these tissues impacts the distribution of leukocyte population, the activity of lymphocytes resulting in a marked change in the overall immune defense [87, 89–91]. Lipid accumulation of lymphoid organs is known to occur in older people and adversely affect their immunity. Consequently, obesity is assumed to promote premature “aging” of the immune system [92]. Also, diet-induced obesity in mice adversely impacts the dynamics of secondary lymphoid tissues leading to alteration of effector/memory T cell ratio and an overall constraint in T cell receptor variety [90, 91, 93]. Therefore, T cells in obese mice are capable of responding to a smaller range of pathogens as compared to the normal chow-fed mice. Obesity also reduces inguinal lymph node size, hampers lymphatic fluid transport, and dendritic cell movement and reduces the number of T lymphocytes in lymph nodes [94]. Overall, obesity disturbs immune system integrity and significantly alters leukocyte growth, movement, and diversity. Indeed, a recent study showed that BMI was inversely correlated with total lymphocyte count in COVID-19 patients [95].

## Insulin resistance negatively impact immune function

Multiple lines of evidence suggest that Insulin may be a key regulator of T-cell metabolism and function [96–99]. Insulin signaling exerts critical immune-stimulatory effects on T-cells, positively controlling their growth and proliferation, glucose metabolism, and production of cytokines, which results in the strengthening of host defense against infections. Obesity often leads to systemic “insulin resistance” a phenomenon that is characterized by reduced insulin signaling in peripheral tissues resulting in several metabolic abnormalities [100–103]. Insulin resistance is a complex phenomenon and multiple factors are involved but obesity induced adipose dysfunction plays a central role in the development of systemic insulin resistance [100–106]. Obesity leads to a significant expansion of adipose mass that radically influences adipose function, which causes disruption of insulin signaling in peripheral tissues including immune cells. Insulin-stimulated signaling pathway is impaired in lymphocytes of individuals with obesity [107] and Type 2 diabetes [108]. Francis M. Finucane and Colin Davenport in a recent paper discussed the possible relationship between insulin resistance with COPVID-19 disease severity [109, 110]. The authors suggested that markers of insulin resistance should be assessed for their prognostic efficacy. No study has specifically looked at the association between insulin resistance and the severity of CoVID-19 disease because clinical and biochemical markers of insulin resistance are not routinely measured in CoVID-19 patients. Ren *et. al*. used triglyceride and glucose index (TyG) as a marker of insulin resistance and showed that TyG index was significantly associated with an increased risk of severe case and mortality in CoVID-19 patients [110]. Although TyG index is a useful surrogate marker, it is not considered a gold standard for assessing insulin resistance. More studies are needed to utilize more acceptable insulin resistance models like Homeostatic Model Assessment (HOMA) or Quantitative insulin sensitivity check index (QUICKI) to investigate the contribution of insulin resistance on disease severity and mortality in CoVID-19 patients [111–113].

## Leptin resistance in obesity impairs immune functioning

Besides insulin, leptin the hormone that is secreted from adipocytes exerts profound effects on innate and adaptive immunity. Leptin is a key regulator of metabolic homeostasis and it primarily exerts its effects via Leptin receptors (LEPR) that are highly expressed in POMC neurons in the hypothalamus, which is the epicenter of appetite and energy expenditure regulation. Leptin has also been shown to regulate several other physiological processes in the body. Interestingly, LEPRs are expressed in cells of the immune system and several studies have documents the role of leptin in regulating various aspects of immune cell development and activity [114–116]. Leptin has been shown to regulate both innate and adaptive immune responses via the modulation of immune cell metabolism, proliferation, and activity. Circulating leptin levels are markedly increased in obese subjects but the response of target tissues to leptin is severely compromised due to leptin resistance [117–119]. Therefore, leptin resistance would profoundly impact the proper development and activity of immune cells in obese subjects, weaken the host defense, and increase the chances of severe disease and poor outcome in COVID-19 patients. A recent paper provided a detailed analysis of the role of leptin in COVID-19 disease severity in obese subjects [120]. The authors describe how leptin plays a vital role in immune regulation and how chronically elevated leptin (as seen in obese subjects) impairs host immune defense. The authors conclude by suggesting studies to explore the possible role of leptin in the pathogenesis of SARS-CoV-2

## Altered ACE2 expression in obese subjects may impact COVID-19 disease severity

Angiotensinogen Converting Enzyme (ACE-2) is required for the entry of COVID-19 into the cells**.** The receptors are expressed on cells in the nose lining, the lungs, pancreas, kidneys and gut, adipose, and in the lining of blood vessels, in the heart muscle, and cells circulating in the blood. It is assumed that increased expression of ACE-2 would boost the entry of the virus into the cells and therefore, cause severe disease with worse clinical outcomes. Emerging evidence indicates that ACE2 expression is increased in individuals who are obese and overweight. Higham et al. have demonstrated increased ACE2 expression in the bronchial epithelium of COPD patients who are overweight or obese compared to lean subjects [121]. The authors suggested that increased ACE-2 expression may be related to increased disease severity in COVID-19 patients who are overweight or obese. Interestingly, ACE-2 expression is higher in adipose compared to lung tissue, which is the primary target of COVID-19 [122]. Moreover, adipose ACE-2 expression is up-regulated in animal models of diet-induced obesity [123]. This raises the prospect of adipose tissue being an important target and a possible reservoir for COVID-19. Adipose tissue has been shown to act as a reservoir for other human pathogens [124]. More importantly, lipid droplets that are present in adipose tissue have been shown to play a key role in the production of the Hepatitis C virus [125–128]. Therefore, it is reasonable to assume that adipose tissues might act as a reservoir for COVID-19 and lipid droplets might facilitate viral production and spread. Consequently, excess adipose as seen in obesity would make it an easy target for the virus entry and spread and therefore, cause severe disease with bad clinical outcomes. More research is needed to understand the functional significance of adipose ACE-2 and its association with obesity in COVID-19 patients.

## Role of coagulopathy /thrombosis in SARS-CoV-2 pathogenesis

Several studies have shown that obesity is associated with a hypercoagulable state and obese subjects have elevated levels of prothrombin factors and reduced levels of anti-thrombin molecules [129–131]. Since, severely ill COVID-19 patients are often associated with coagulopathy/thrombosis and obesity could potentially make it worse. A study by Gazzaruso et al. on a cohort of 49 patients hospitalized with COVID-19 infection and reported that low antithrombin (AT) levels were strongly associated with increased mortality [132]. The authors further show that BMI was the only variable that showed a significant difference between patients with low and high levels of AT. The authors documented an inverse correlation between AT levels and BMI and obese patients had significantly lower AT levels as compared to non-obese patients. The authors suggested that AT may be the connecting factor behind increased mortality in obese COVID-19 patients. More studies are needed to confirm this finding.

## Does obesity survival paradox occur in COVID-19 patients?

Obese subjects are at an increased risk of developing pneumonia but ironically, obese patients with pneumonia have a lower mortality as compared to non-obese subjects. This phenomenon is known as “Obesity survival paradox” and has been the subject of several independent studies [133–136]. Obesity survival paradox in COVID-19 patients is still a matter of debate. Biscarini et al. analyzed a cohort of 331 patients admitted to hospital with COVID-19. The authors reported that obese COVID-19 patients were more likely to be admitted to ICU than non-obese subjects but obesity was not significantly associated with mortality, mortality in ICU and length of hospital stay [137]. However, majority of the studies have reported that obese subjects are an increased risk of severe disease and increased mortality due to COVID-19 [59, 132, 138–143].

## Conclusion

**Fig. 2:**
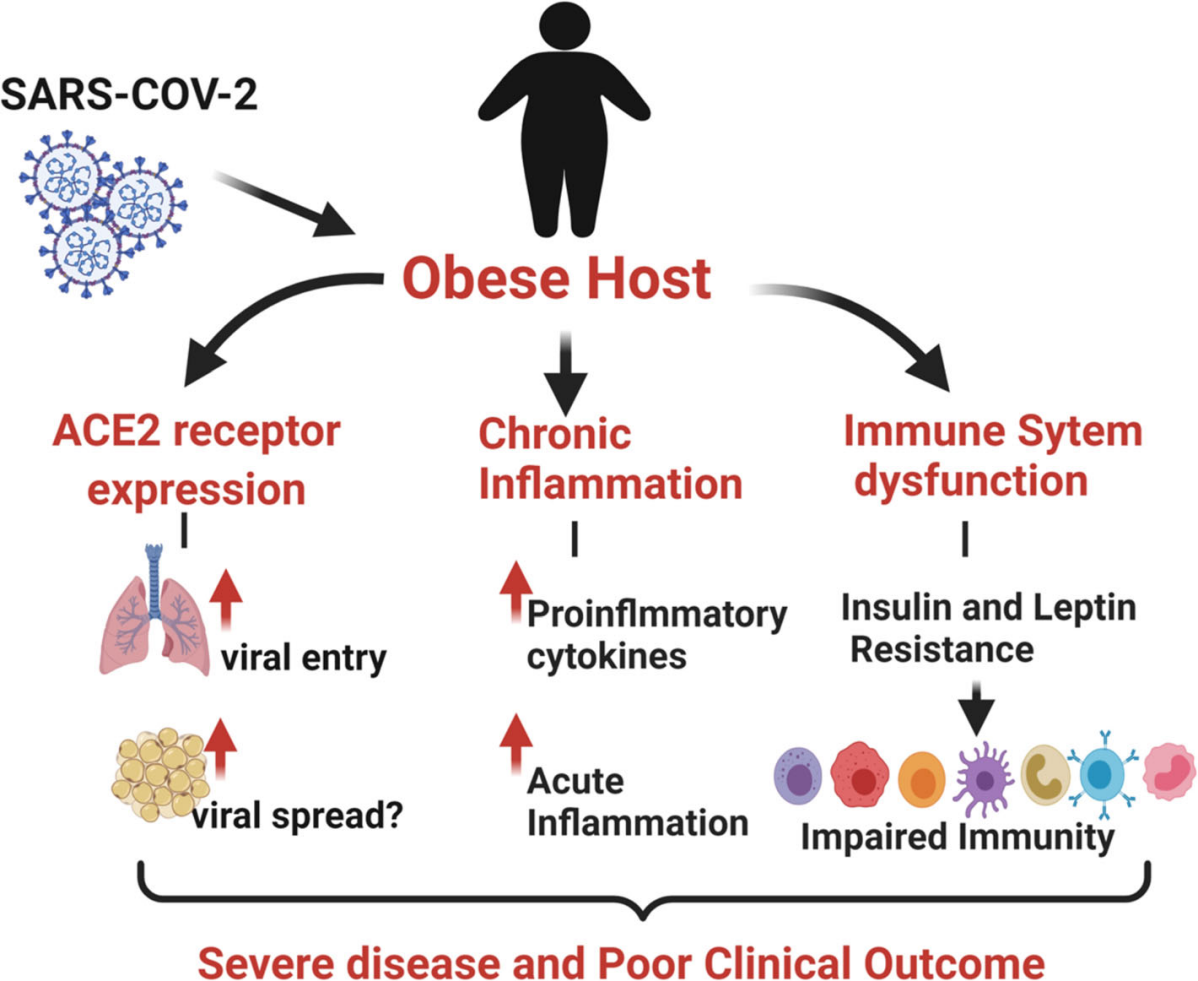
Factors responsible for disease severity and poor outcome in obese COVID-19 patients. Obesity-associate chronic inflammation, impaired Immune function and increased ACE2 expression results in an increased disease severity and worse clinical outcome in obese subjects with COVID-19 infection

Obesity is a huge healthcare concern because it is associated with several chronic diseases including type 2 diabetes, heart diseases, stroke, and certain types of cancers. Obesity significantly reduces the quality of life and is one of the leading causes of death, worldwide. Recent evidence has shown that obesity weakens the immune system and therefore, making the host vulnerable to infectious diseases. Indeed, Obesity has emerged as a strong risk factor for severe disease in the current pandemic disease, COVID-19. Several independent studies have demonstrated that obese subjects with COVID-19 have a higher risk of severe disease, hospitalization, and increased probability of death. During the 2009 HIN1 pandemic, patients with severe obesity were more likely to require hospitalization, ICU admission, and death due to the disease. Data over the years have indicated that obesity negatively impacts host immune defense making it vulnerable to infectious disease. Excess adiposity is associated with significant changes in the resident immune cell composition of adipose tissue, which disrupts the balance between pro-inflammatory and anti-inflammatory immune cells in favor of the former. This leads to a state of chronic low-grade inflammation. This chronic inflammation is likely amplified by acute inflammation arising out of COVID-19 resulting in a more severe disease phenotype and poorer outcomes. One the other hand excess lipid deposition alters the integrity and architecture of primary lymphoid tissues and thereby impacting the immune cell development and activation. Besides, metabolic changes associated with obesity such as insulin and leptin resistance negatively impact immune cell function. Together these changes have a substantial influence on immune cell growth and proliferation, glucose metabolism, and activation which ultimately results in impairment of host immune defense. Finally, adipose ACE-2 could also play a vital role in the spread of COVID-19 to other tissues but more work is needed to investigate this possibility. Fig. 2 illustrates the possible mechanism(s) that could explain increasing susceptibility of the obese subject to severe disease and poor clinical outcome as a result of COVID-19 infection.

## Data Availability

Agreed.

## Abbreviations

SARS-COV- 2: Severe acute respiratory syndrome coronavirus 2
COVID-19: Coronavirus disease
BMI: Body Mass Index
IMV: Intermittent Mandatory Ventilation
ICU: Intensive Care Unit
MAFLD: Metabolic associated fatty liver disease
H1N1: Influenza A virus subtype H1N1
ACE-2: Angiotensin-converting enzyme 2
T_h_ cells: T helper cells
T-_Reg_ cells: Regulatory T cells
TLR: Toll-like receptors
TCR: T-cell receptor
INSR: Insulin receptor
LEPR: Leptin receptor
POMC: Pro-opiomelanocortin
